# Expected and unexpected effects after systemic inhibition of Hippo transcriptional output in cancer

**DOI:** 10.1038/s41467-024-46531-1

**Published:** 2024-03-27

**Authors:** Isabel Baroja, Nikolaos C. Kyriakidis, Georg Halder, Iván M. Moya

**Affiliations:** 1 Cancer Research Group, Faculty of Engineering and Applied Sciences, Universidad de Las Américas, Quito, Ecuador; 2 Faculty of Health Sciences and Medicine, Universidad de Extremadura, Mérida, Spain; 3 Cancer Research Group, Faculty of Health Sciences, Universidad de Las Américas, Quito, Ecuador; 4 VIB Center for Cancer Biology and Department of Oncology, KU Leuven, Leuven, Belgium

**Keywords:** Molecular medicine, Targeted therapies

## Abstract

Hyperactivation of YAP/TAZ, the Hippo pathway downstream effectors, is common in human cancer. The requirement of YAP/TAZ for cancer cell survival in preclinical models, prompted the development of pharmacological inhibitors that suppress their transcriptional activity. However, systemic YAP/TAZ inhibition may sometimes have unpredictable patient outcomes, with limited or even adverse effects because YAP/TAZ action is not simply tumor promoting but also tumor suppressive in some cell types. Here, we review the role of the Hippo pathway in distinct tumor cell populations, discuss the impact of inhibiting Hippo output on tumor growth, and examine current developments in YAP/TAZ inhibitors.

## Abstract (web summary)

Hyperactivation of YAP/TAZ, the Hippo pathway downstream effectors, is common in human cancer. In this perspective, the authors review the role of the Hippo pathway in distinct tumor cell populations, discuss the impact of inhibiting Hippo output on tumor growth, and examine current developments in YAP/TAZ inhibitors.

## Introduction

The Hippo pathway is a signal transduction pathway (Box 1A) that has emerged as a potential target to treat a variety of cancers. This is because the Hippo pathway is widely deregulated in cancer cells and is associated with poor prognosis in different types of human cancer^1,2^. Mutations in Hippo pathway components, such as amplifications of *YAP* or loss of function mutations in *NF2*, *LATS1*, and *LATS2*, are frequently present in many cancer types^1,2^. However, hyperactivation of YAP/TAZ in cancer is present at much higher frequency than this, probably caused by non-genetic mechanisms^1,2^. Hippo pathway deregulation leads to nuclear localization and hyperactivation of YAP and TAZ, which promote tumor growth by driving a transcriptional program that induces cancer cell plasticity, resistance to cellular stresses, avoidance of immune surveillance, and metastatic behavior (Box 1B)^1,2^. Genetic experiments in human cancer cells and in mouse cancer models showed that YAP/TAZ activity is often required for tumor initiation and progression^1,2^. Thus, YAP/TAZ are attractive targets for cancer therapy because they are required for the development of different types of cancer in many organs.

Several small molecule inhibitors that block the function of the YAP/TAZ-TEAD transcription factor complexes were recently developed and some of them are currently being tested in clinical trials for cancer therapy. YAP/TAZ-TEAD inhibition triggered tumor regression in preclinical mouse models for mesothelioma^3–7^ and first data report clinical benefits for mesothelioma patients and patients with NF2 mutant sarcoma: ION537 (NCT04659096), VT3989 (NCT04665206), IK-930 88 NCT05228015) and IAG933 (NCT04857372). It is expected that YAP/TAZ-TEAD inhibition also eliminates cancers with driver mutations in Hippo pathway components such as mesothelioma, meningioma, renal cell carcinoma and cholangiocarcinoma and cancers where YAP/TAZ are hyperactivated but that do not have driver mutations in Hippo pathway components^1,2,8^. However, while it is expected that YAP/TAZ-TEAD inhibitors may offer broad therapeutic opportunities, it is currently not known whether patients without driver mutations in Hippo pathway components will benefit from YAP/TAZ-TEAD inhibitor treatment. In fact, predicting the effects of systemic YAP/TAZ-TEAD inhibition by a small molecule inhibitor is not trivial. Systemic inhibition blocks YAP/TAZ not only in tumor cells but in all cells of the body, which may trigger outcomes that are not simply predicted from genetic experiments where YAP/TAZ-TEAD were specifically inactivated in tumor cells. For example, if YAP/TAZ are required for a tumor-suppressing activity in tumor-associated cells, then systemic YAP/TAZ-TEAD inhibition may promote tumor growth even if YAP/TAZ are active in tumor cells. In this article, we review our current understanding of the role of the Hippo pathway in distinct tumor and tumor-associated cells and discuss potential challenges and outcomes of systemic YAP/TAZ-TEAD inhibition.

Box 1 The Hippo signaling pathway

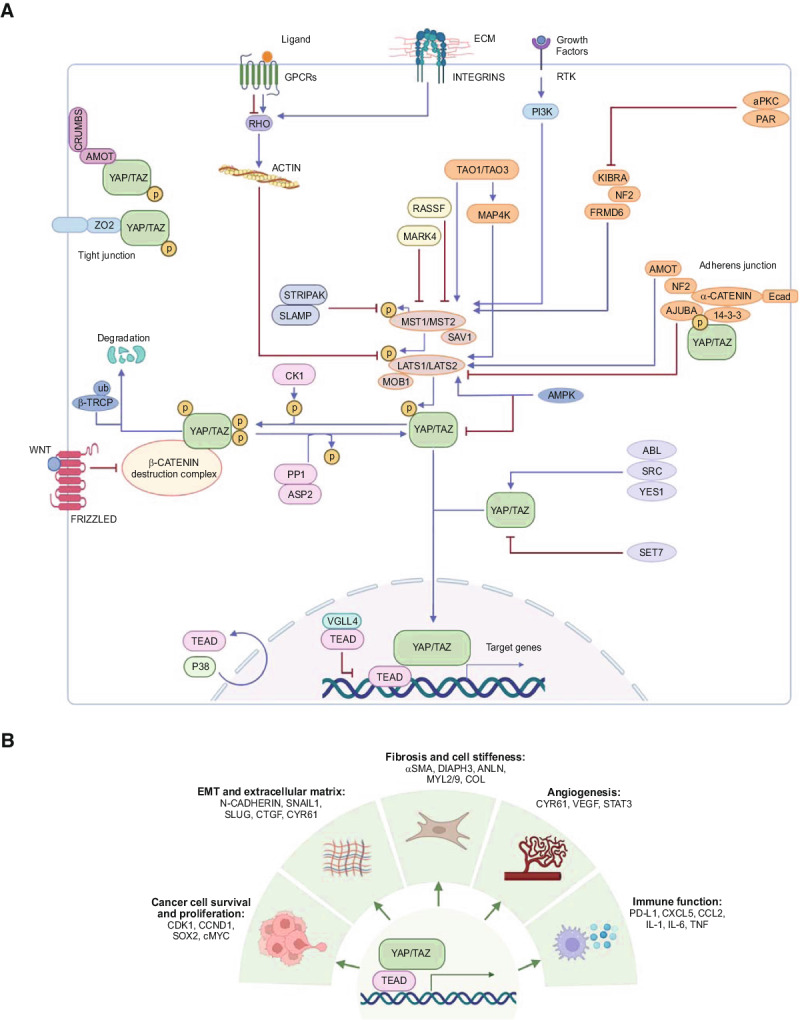

The Hippo pathway is a signal transduction pathway that integrates extra- and intracellular signals into transcriptional programs that modulate cell fate, function, and survival^95,96^. **A** At the core of the Hippo pathway is a kinase cascade composed of the mammalian Ste20-like kinases 1 and 2 (MST1 and MST2), the mitogen-activated protein kinase 4 (MAPK4) family, the large tumor suppressor kinases 1 and 2 (LATS1 and LATS2), the homologous Yes-associated protein (YAP) and transcriptional co-activator with PDZ-binding motif (TAZ), and the TEA domain family of transcription factors (TEAD1-4). Activation of the Hippo pathway inhibits the transcriptional output of the pathway and depends on the phosphorylation of the core Hippo kinases, MST1/2, and LATS1/2. When MST1-2/MAPK4 are active, they phosphorylate and activate the LATS kinases, which in turn phosphorylate and inactivate YAP and TAZ. The MST1/2 autophosphorylation can be repressed by the STRIPAK–SLMAP protein phosphatase 2 A (PP2A) complex. MST1/2 are also activated by TAO kinases, whereas LATS1/2 can also be phosphorylated by MAP4Ks. Activation of LATS1/2 induces the phosphorylation of YAP and TAZ and inhibits their transcription co-activator function. Phosphorylated YAP/TAZ are exported from the nucleus and degraded in the cytoplasm or sequestered at cellular junctions. In contrast, when the core kinases are inactive, YAP and TAZ translocate into the nucleus where they act as transcriptional co-activators by binding to TEAD and other transcription factors and regulate the expression of target genes. The activity of the core components of the Hippo pathway is regulated by multiple mechanisms, such as mechanical forces and integrin signaling from the extracellular matrix (ECM) that modulate the actin cytoskeleton; mechanisms involved in establishing apical-basal cell polarity, including the Crumbs complex and the aPKC–PAR complex; the use of scaffolding proteins located at cell junctions such as angiomotin (AMOT), neurofibromin 2 (NF2; also known as Merlin), kidney and brain protein (KIBRA; also known as WWC1), AJUBA, and zonula occludens (ZO) proteins; and receptor tyrosine kinases (RTKs) and G protein-coupled receptors (GPCRs) that act at the cell surface. Also, Hippo pathway components can interact with components of other pathways, such as the WNT signaling pathway, where the β-Catenin destruction complex associates with YAP/TAZ that are then targeted for β-TrCP-mediated degradation. In addition, metabolic inputs are relayed to Hippo signaling via AMP-activated protein kinase (AMPK). **B** YAP and TAZ regulate target genes that drive diverse processes depending on the cell type where they are activated. P, phosphorylation; RASSF, RAS association domain family; Ub, ubiquitylation.

## YAP/TAZ-TEAD as promoters of tumor growth

**Fig. 1:**
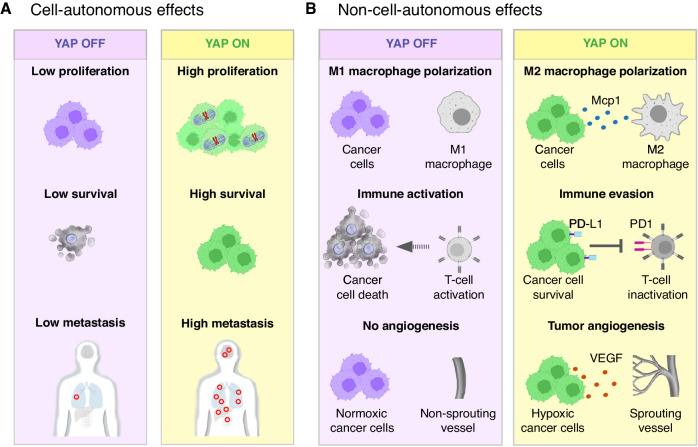
Tumor promoting functions of YAP/TAZ in cancer cells. **A** YAP and TAZ are frequently activated in cancer cells, where they exert tumor promoting functions. In most types of cancer cells, their activation promotes cancer progression by stimulating cell proliferation, survival, and migration. **B** YAP/TAZ activation in cancer cells can also promote tumor growth by non-cell autonomously modulating the activity of tumor associated cells. YAP and TAZ in cancer cell can indirectly suppress the differentiation and activation of cytotoxic T-cells cells and the recruitment and differentiation immune suppressive cells such as Treg and tumor-associated M2-like macrophages by regulating the expression of immune modulatory signaling proteins. Also, YAP and TAZ activation in cancer cells promotes the expression of pro-angiogenic factors, such as VEGF, leading to the formation of new blood vessels that supply nutrients and oxygen to the growing tumor. Thus, tumor growth is orchestrated by cell autonomous and non-cell autonomous effects of YAP/TAZ in cancer cells. PD1, Programmed Cell Death 1; PD-L1, Programmed Cell Death 1 Ligand 1.

**Fig. 2:**
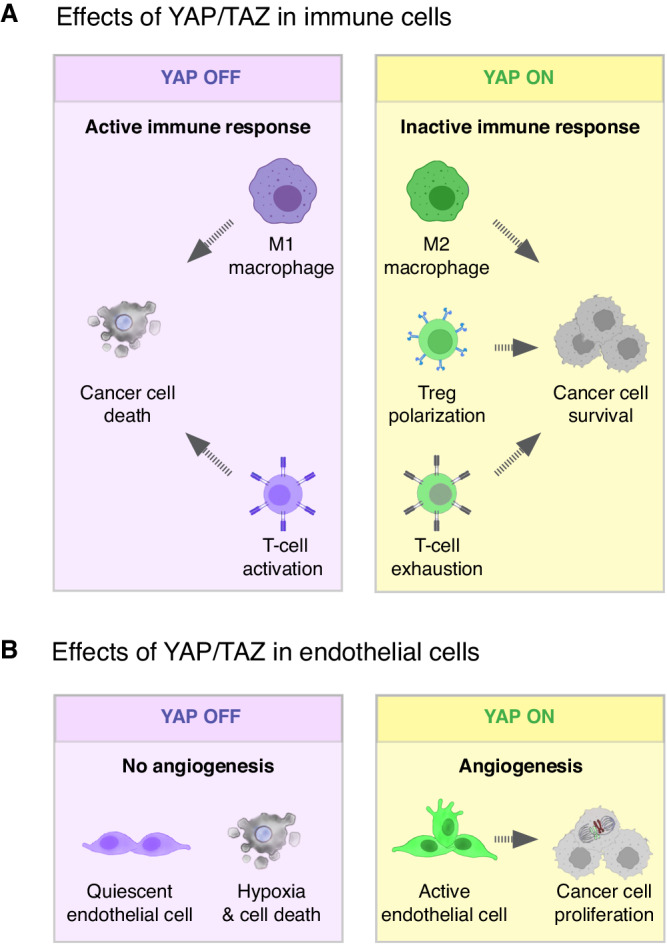
Tumor promoting functions of YAP/TAZ activity in tumor-associated cells. **A** YAP and TAZ can induce tumor growth by promoting the recruitment and differentiation of immune suppressive cells such as tumor-associated M2-like macrophages and Tregs or by inhibiting the differentiation and activation of cytotoxic T-cells. **B** YAP and TAZ activation in endothelial cells stimulates their migration and proliferation, which is required for tumor angiogenesis. Therefore, inhibiting YAP and TAZ in endothelial cells could potentially decrease tumor growth by reducing tumor angiogenesis, which would deplete the supply of nutrients and oxygen to the tumor. Treg, regulatory T-cell.

Activated YAP/TAZ are often promoters of tumor growth, and may do so in three different ways: First, YAP/TAZ may activate genes that promote cell proliferation, survival and other cancer hallmarks directly in cancer cells (Fig. 1A). Second, YAP/TAZ target genes may non-cell autonomously activate pro-tumoral functions in non-cancer cells, such as immune cells, and endothelial cells (Fig.1B). Third, activation of YAP/TAZ in non-cancer cells may modulate their behavior to suport tumor growth (Fig. 2A, B). Inhibition of YAP/TAZ-TEAD in all these cases will cause tumor regression by blocking tumor promoting functions in cancer cells and non-cancer cells.

### Tumor-promoting functions of YAP/TAZ in cancer cells

Deregulation of the Hippo pathway has been observed in a variety of human cancers. Genetic inactivation of *Yap/Taz* strongly reduces the number of tumors in mouse models for liver cancer, colorectal cancer, non-small-cell lung cancer, breast cancer, gastric cancer, pancreatic adenocarcinoma, and glioblastoma^1,2^. In those cancers, hyperactivation of YAP and TAZ induces a transcriptional program that includes target genes involved in cell cycle progression, DNA repair, cell survival, and cell migration. Key target genes include *CDK1* and *CCND1*, which direct cell cycle progression, and synthesis and repair of DNA^9^; *c-MYC*, which promotes tumor initiation and progression^10^; *SOX2*, which confers cancer stem cell traits^11^; *AXL*, *BIRC5* and *BCL2*, which promote cancer cell survival and metastasis^12,13^; *CCN1* (CYR61), which regulates cell proliferation and angiogenesis^14^; *CCN2* (connective tissue growth factor, CTGF), which regulates cell adhesion and migration^15^; and *CDH2* (*N-CADHERIN*), *SNAI1* and *SNAI2*, (SLUG) which promote metastatic potential and induce epithelial-to-mesenchymal transition^16^. These and other YAP/TAZ target genes promote cancer development and position the Hippo pathway as a prime candidate for targeted therapy against cancer. Thus, inhibiting YAP/TAZ may suppress those cancer cell traits and cause tumor regression.

### YAP/TAZ in cancer cells induces pro-tumoral immune cell activity

YAP/TAZ activity in cancer cells can indirectly promote tumor growth by modifying the action of immune cells present in the tumor microenvironment, which can be coerced to nurture cancer cells (Fig.1B). For example, the expression of YAP/TAZ in liver, colon and prostate cancer cells affected the recruitment and polarization of immunosuppressive M2 macrophages^17–19^. In the mouse liver, activation of YAP/TAZ in hepatocytes by deletion of *Mst1* and *Mst2* or by overexpression of *Yap* caused liver inflammation and promoted macrophage infiltration in addition to their classic effects of inducing hepatocyte proliferation and liver cancer^18–20^. Mechanistically, YAP/TAZ drive the expression o *Ccl2*, which encodes for monocyte chemoattractant protein 1 (MCP1) and *Csf1*, wich encodes for colony stimulating factor 1 (CSF1). These signaling proteins trigger the infiltration of macrophages with mixed M1 and M2 phenotypes, which boosted immune cell evasion, clonal expansion, and tumor growth. In addition, YAP/TAZ activation in human cancer cells facilitated the evasion of adaptative immunity by promoting T-cell exhaustion through inducing *PD-L1* expression (Fig.1B)^21,22^. Collectively, these studies indicate that the tumor-promoting effect of YAP/TAZ activation in cancer cells includes the induction of signaling molecules that modulate the behavior of immune cells and generate a tumor-promoting environment. Thus, in addition to the direct effects on cancer cells, inhibition of YAP/TAZ-TEAD may indirectly enhance the antitumoral surveillance activity of different immune cells.

### Activation of YAP/TAZ in cancer cells recruits endothelial cells

Tumor growth relies on proper vascularization for the delivery of oxygen and nutrients. YAP/TAZ promotes angiogenesis and tumor growth by inducing the expression of vascular endothelial growth factor (*VEGFA*) and other genes encoding for secreted pro-angiogenic factors in hypoxic cancer cells (Fig.1B)^23^. Knockdown of *YAP* in renal carcinoma cells decreased the levels of VEGF expression, which resulted in poor recruitment of endothelial cells in vitro and low induction of tumor angiogenesis in vivo^24^. Therefore, inhibiting YAP/TAZ-TEAD in cancer cells dampens paracrine pro-angiogenic signaling and presents a strategy to suppress the formation of new blood vessels. This may be clinically important because targeting VEGF and its receptors has limited therapeutic efficacy, since the most common anti-VEGF drug (bevacizumab) blocks the binding of VEGF to receptor tyrosine kinases (VEGFRs) but not to its alternative coreceptors Neuropilin 1 and 2 (NRP1/2), which can transduce VEGF signaling in the absence of VEGFR^25,26^. Thus, inhibiting YAP/TAZ-TEAD offers the possibility to reduce VEGF production and signaling without needing to inhibit several redundant VEGF receptors.

### Tumor-promoting function of YAP/TAZ in macrophages

M2 macrophages suppress immune clearance, stimulate angiogenesis, and promote cancer cell proliferation. Experimental YAP/TAZ activation in human macrophages induced a pro-tumor M2-like phenotype (Fig.2A)^27^. Thus, YAP activation increased the expression of M2-associated markers such as *IL-10, TG, mFB1,* and *VEGFA* but decreased the M1 marker *IL1B*^28^. Conversely, silencing *YAP* expression in cultured human monocytic cells, which are undifferentiated macrophage precursor cells, reduced the levels of the M2 markers interleukin 4 (*IL-4*), transforming growth factor B, *β1 (TGFB)*, and chitinase-like protein Ym2 (CHIA), while it increased the expression of nitric oxide synthase (*NOS2*), which is a M1 marker^29^. Interestingly, cancer cells can activate YAP in macrophages to enhance their microenvironment: triple-negative human breast cancer cells caused the activation of YAP in co-cultured macrophages, which in turn induced M2 polarization that then promoted cancer cell metastasis and decreased mouse survival in xenograft models^28^. Therefore, inhibition of YAP/TAZ activity in macrophages and their precursors may promote M1-like macrophage polarization and dampen tumor growth by activating their antitumoral activity.

### YAP activation in T-cells inhibits adaptive immunity

Inhibition of YAP can affect tumor growth by influencing the development and function of different types of T-cells (Fig.2A). First, YAP (but not TAZ) is required for the activation, differentiation, and effector function of regulatory T-cells (Treg)^30,31^. Tregs are a specialized subpopulation of T-cells that suppress immune responses and inhibit T-cell proliferation and antitumor immunity. Deletion of *Yap* in mouse Tregs reduced *Acvr1c* expression and the effectiveness of ACVR1C protein in suppressing the proliferation and function of naïve CD4^+^ T-cells, indicating that YAP promotes the immune suppressive function of Tregs^31,32^. Second, high levels of YAP in T-cells is correlated with decreased survival in diverse human cancers because YAP inhibits cytotoxic T-cell differentiation and induces a dysfunctional or exhausted state^33^. Deletion of *YAP* in human cultured CD8^+^ T-cells enhanced their cytotoxic activity and their ability to kill *OVA*-expressing melanoma tumor cells (B16F10)^30^, and deletion of *Yap* in mouse effector T-cells induced T-cell activation, differentiation and enhanced their ability to infiltrate and eliminate highly immunosuppressive melanoma and Lewis lung carcinomas^33^. Mechanistically, *Yap* deletion caused the upregulation of beta-lymphocyte-induced maturation protein 1 (BLIMP1), which is an inducer of terminal T-cell differentiation, and elevated the production of interferon gamma (IFNγ) and tumor necrosis factor alpha (TNFα) which cause anti-tumor effects^34^. Thus, systemically inhibiting YAP-TEAD may enhance the antitumoral function of T-cells while blocking the immune-suppressing effect of Tregs^30,31^.

### YAP/TAZ activation in fibroblasts promotes tumor growth

Cancer-associated fibroblasts (CAFs) are active components of the tumor microenvironment that can promote tumor growth by remodeling the extracellular matrix and by modulating angiogenesis, immune cell function, cancer cell metastasis, proliferation, and drug resistance. YAP plays a key role in the conversion of quiescent fibroblasts into activated fibroblasts in various normal and malignant tissues^35,36^. In the liver, resident fibroblasts, so-called stellate cells, have low YAP/TAZ activity, but require YAP/TAZ activation to transition into an activated, pro-fibrotic and pro-inflammatory state in chronic liver disease^37^. Analogously, in breast and prostate cancer tissues, activated fibroblasts show strong nuclear YAP, while YAP localizes to the cytoplasm in quiescent and inactivated fibroblasts^35,36^.

Experimental YAP/TAZ activation in fibroblasts is sufficient to promote a stiffer and pro-fibrotic microenvironment that potentiates tumor growth. Overexpression of human *YAP* in mouse fibroblasts induced the expression of the pro-fibrotic mediator genes *Interleukin 11 IL11, Connective tissue growth factor (CTGF), Cysteine-rich angiogenic inducer 61 (CYR61)*, *COL1A1* (collagen) and *LAMA1* (laminin), and intracellular regulators of the cytoskeleton, such as smooth muscle actin (*ACTA2*), diaphanous related formin 3 (DIAPH3), anillin actin binding protein (*ANLN*) and myosin light chain 2 and 9 (*MYL2/9*)^35^. The cumulative action of these genes results in the activation of fibroblast and in the stiffening of the surrounding matrix. When YAP-activated fibroblasts were xenotransplanted together with prostate cancer cells into mice, cancer cells proliferated more and tumors grew larger than in mice where cancer cells were xenotransplanted alone or with fibroblasts where *YAP* expression was silenced by *shRNA*^36^. Similarly, in subcutaneous xenograft models of colorectal cancer, downregulation of the upstream component of the Hippo pathway MOB1A in CAFs, activated YAP/TAZ in CAFs, which in turn promoted cancer cell proliferation and tumor growth^38^. In contrast, experimental depletion of *YAP* in CAFs by siRNA knockdown in mouse models of human breast cancer and squamous cell carcinoma reduced their ability to form fibrous collagen networks, induce angiogenesis, and promote tumor growth and metastasis^35^. Thus, YAP activation in CAFs is a general feature of diverse cancers. Therefore, systemic inhibition of YAP may block CAF activation, reduce the stiffness of the tumor microenvironment, and non-cell autonomously restrict the proliferation and survival of a variety of cancer cells.

### YAP/TAZ activation in endothelial cells drive angiogenesis

YAP/TAZ are required in endothelial cells for the development of new blood vessels in normal and malignant tissues (Fig. 2B)^39–41^. In the developing vasculature of the mouse retina, for example, YAP and TAZ are expressed in all endothelial cells, but are mainly activated at the angiogenic front, the zone of the retina where new vessels form^39–41^. Once activated in endothelial cells, YAP/TAZ drive the expression of target genes that promote endothelial cell proliferation, cell adhesion, migration, and cytoskeleton remodeling^40,42^. VEGF is a major inducer of angiogenesis, and it signals through VEGF receptors (VEGFR) that activate YAP/TAZ transcriptional activity and endothelial cell sprouting^40,42^. Interestingly, *VEGFA* is a YAP-target gene in cancer cells^24^ and experimental activation of YAP in mouse endothelial cells of transplanted Lewis lung carcinoma promoted tumor growth and progression by enhancing angiogenesis and tumor cell invasion^42^. Therefore, YAP induces angiogenesis by two mechanisms: First, YAP activation in cancer cells induces the production of VEGF (Fig.1B) and second, YAP activation in endothelial cells triggers angiogenesis (Fig.2B). The central role of YAP/TAZ for the activation and function of VEGF signaling highlights the potential of targeting YAP/TAZ activity to modulate normal and tumor angiogenesis.

Tumor growth in mouse models depends on the activation of YAP/TAZ in endothelial cells and the induction of tumor angiogenesis^42–44^. For example, pharmacological inhibition of YAP/TAZ-TEAD function by Verteporfin suppressed in vitro angiogenesis in coculture assays of human endothelial cells with esophageal squamous carcinoma cells^44^ or with pancreatic ductal adenocarcinoma cells^43^. In vivo, deletion of *Yap/Taz* or *Tead1,2,4* in endothelial cells stunted vascular growth in mice^40,45^. In cancer, inhibition of YAP/TAZ with Verteporfin or genetic ablation of *Yap/Taz* in endothelial cells reduced vessel density and tumor progression in mouse models of colorectal cancer and melanoma^46^. Similarly, Verteporfin suppressed angiogenesis and vasculogenic mimicry in xenograft models of pancreatic ductal adenocarcinoma via suppressing *Angpt2*, *Mmp2*, *Cdh5*, and *Acta2* expression^43^. Thus, systemic inhibition of YAP/TAZ can target tumor endothelial cells and may be a promising therapeutic approach for treating several types of cancer.

Current YAP/TAZ inhibitors mainly target the binding of YAP/TAZ to TEAD; yet, in addition, or in parallel to TEAD proteins^45^, the pro-angiogenic function of YAP/TAZ are mediated by binding to signal transducer and activator of transcription 3 (STAT3)^42,46,47^. YAP/TAZ can form large complexes with STAT3, TEAD, and AP-1 in other cell types^48^, yet whether YAP/TAZ bind to STAT3 and TEAD independently or whether all these proteins form a large complex in endothelial cells is not known. However, genetic ablation of mouse *Stat3* in endothelial cells inhibited tumor angiogenesis and the growth of colorectal cancer and melanoma in mice, thus phenocopying the effects of *Yap/Taz* deletion^46^. Therefore, the individual deletion of any of these genes resulted in similar phenotypes in endothelial cells, implying that YAP/TAZ, TEAD and STAT3 collaboratively induce angiogenic growth. Thus, while current inhibitors blocking YAP/TAZ-TEAD function might be effective against tumor angiogenesis, a more potent inhibition of tumor angiogenesis may be achieved by blocking complex formation between YAP/TAZ, AP1, and STAT3.

Altogether, the studies presented in this section illustrate that YAP/TAZ can not only promote tumor growth and cancer progression when they are activated in cancer cells but also when they are activated in diverse cancer associated cell types. These results imply that systemic inhibition of YAP/TAZ in all cells may cause more dramatic anti-tumor effects than would be predicted based on effects when *YAP/TAZ* were specifically deleted in cancer cells or another cell population. For example, it is conceivable that systemic YAP/TAZ inhibition may cause regression of tumors for which *YAP/TAZ* deletion in tumor cells specifically had no effect on tumor growth.

## YAP/TAZ as suppressors of tumor growth

In contrast to the examples discussed above, in this section, we discuss cases where YAP/TAZ act as tumor suppressors and where their systemic inhibition may promote tumor growth.

### Tumor suppressing functions of YAP/TAZ in cancer cells

**Fig. 3:**
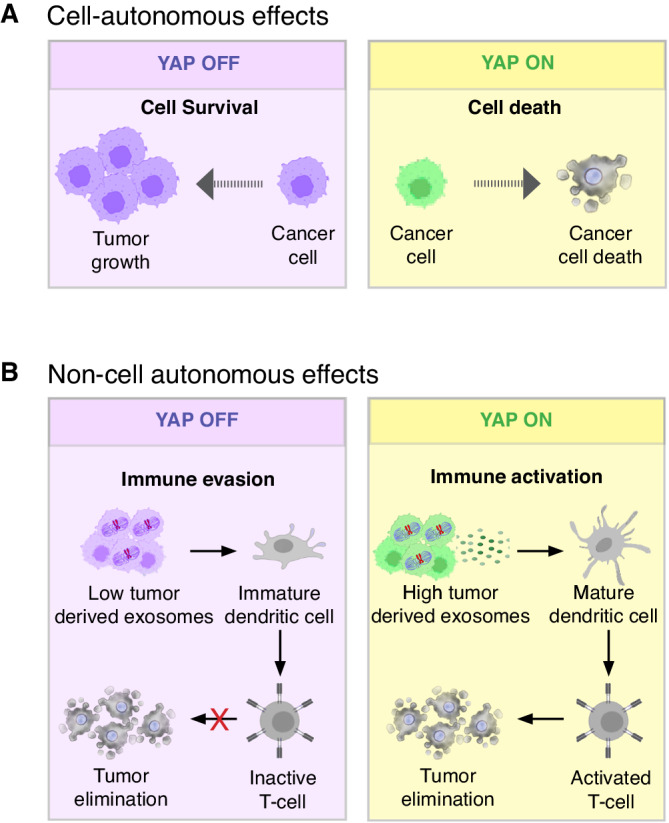
Tumor suppressive functions of YAP/TAZ in cancer cells. **A** YAP/TAZ activation in cancer cells can also cell autonomously trigger tumor suppression. This is because in some types of cancers, such as breast, colon and hematological cancers, YAP/TAZ activation induces cancer cell death. **B** YAP/TAZ activation in cancer cells can inhibit tumor growth by non-cell autonomously activating tumor suppressive T-cells. This is because YAP/TAZ activation in cancer cells promotes the production and secretion of exosomes that stimulate antigen presenting cells and activate T-cells in the tumor microenvironment.

While YAP and TAZ typically promote the proliferation of cancer cells and tumor growth, there are cases in breast, prostate, lung, and colon cancers, where their activation in cancer cells suppresses cell proliferation and survival (Fig. 3A)^49–53^. In luminal breast cancer, for instance, high levels of YAP expression are correlated with better patient survival due to YAP-induced cell death^54^. Hyperactivation of YAP in cultured MCF7 cells, an ER^+^ luminal-like breast cancer cell line, induced the expression of the pro-apoptotic genes encoding for PUMA, BAX and p53AIP1^49^, and experimental hyperactivation of YAP in ER^+^ breast tumors in vivo inhibited the ERα transcriptional program and caused cell death^50,54^. Similarly, in patient-derived xenografts and mouse models of primary and metastatic colon cancer, the experimental hyperactivation of YAP in cancer cells suppressed tumor growth even when WNT signaling was constitutively activated by the deletion of *Apc* (Fig.3A)^52,53^. Such tumor suppressor effects of YAP/TAZ were not limited to solid malignancies as YAP/TAZ activation in myeloma, lymphoma, and leukemia caused cellular stress that triggered DNA damage and induced apoptosis^55,56^.

These examples indicate that inhibiting YAP/TAZ activity in some cancers may promote tumor growth rather than cause tumor regression. Indeed, loss of *YAP* in human colorectal cancer cells is correlated with increased cancer cell proliferation, the development of higher-grade tumors, and worse patient prognosis^52,53^. Similarly, YAP and TAZ are often inactivated in hematological cancer cells due to gene deletions or transcriptional and posttranscriptional repression mechanisms^55,56^. In mouse models of xenotransplanted human breast cancer cells, the genetic inactivation of *YAP/TAZ* reduced the survival of mice and promoted tumor growth and progression^50^.Altogether, these findings suggest that YAP/TAZ may restrain tumor growth in some cancers, and that inhibition of YAP/TAZ-TEAD could worsen some patient outcomes.

### YAP/TAZ activate antitumoral immune surveillance

High YAP/TAZ activity in cancer cells can induce tumor suppressing immune surveillance in some contexts (Fig.3B). Activation of YAP/TAZ by loss of the Hippo pathway kinases *Lats1* and *Lats2* in tumor cells of different murine syngeneic tumor models of melanoma, head and neck carcinoma, and breast cancer enhanced anti-tumor immune responses that lead to cancer cell elimination^57^. This was because the activation of YAP/TAZ induced the secretion of extracellular vesicles that mobilized the innate immune system to mount a strong anti-tumor response (Fig.3B)^57^. Such extracellular vesicles, known as exosomes, were loaded with nucleic-acids from cancer cells and induced a type I interferon response in cytotoxic T-cells and B-cells by activation of the endogenous nucleic-acid-sensing pathways through Toll-like receptor signaling^57^. Interestingly, YAP/TAZ activation by *Lats1/2* deletion not only led to the elimination of existing tumors but induced a long-lasting recognition of tumor cells by the adaptive immune system^57^. Thus, YAP/TAZ activation in cancer cells induced a persistent “vaccine-like” anti-tumor immunogenic response that protected mice from subsequent tumor outgrowths^57^. This implies that inhibiting YAP/TAZ-TEAD in some cancers driven by Hippo pathway mutations may potentially enhance tumor growth by reducing their immunogenicity. However, this may not be a problem if the immune system was already trained before the start of the treatment.

### Tumor suppressing functions of YAP/TAZ in immune cells

**Fig. 4:**
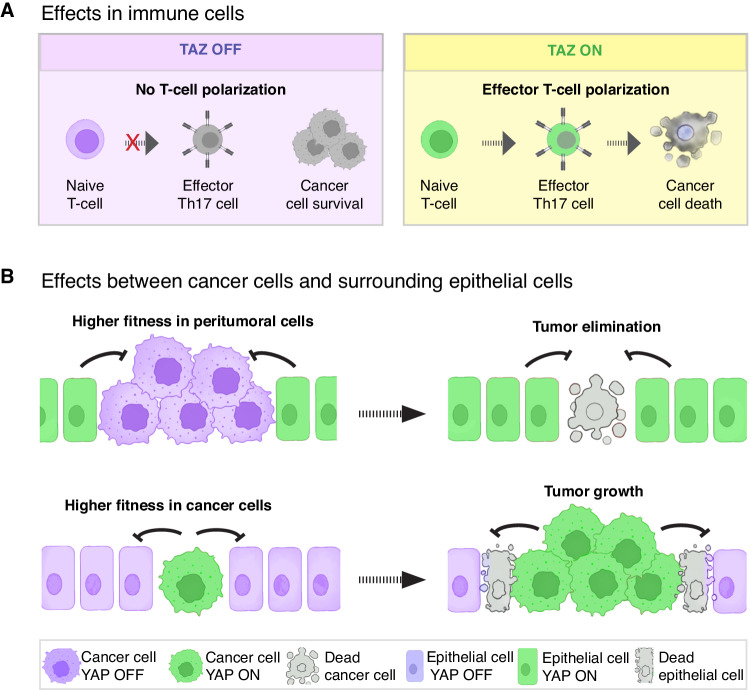
Anti-tumor functions of YAP/TAZ in non-cancer cells. **A** TAZ activation in naïve T-cells potentiates the differentiation and function of effector T-cells, leading to cancer cell elimination. **B** YAP and TAZ elevate the fitness of cells in cell competition, a process by which cells within a tissue compete for survival and growth. Higher levels of YAP/TAZ activation confer a selective advantage to cells (“winner” cells), promoting their growth and proliferation, which can contribute to tumorigenesis, while lower levels of YAP/TAZ activation reduce the competitive potential of cells and turn them into “loser” cells. Depending on the levels of YAP/TAZ activation, both tumor cells or normal surrounding cells can acquire either winner or loser phenotypes. Therefore, the tumor promoting and the tumor suppressing functions of YAP/TAZ in cell competition thus depend on the specific context and the cellular compartment in which they are activated.

Inhibiting YAP/TAZ-TEAD can favor tumor growth by suppressing neutrophil and T-cell anti-cancer activities^58,59^. Experimental hyperactivation of YAP/TAZ in mouse neutrophils induced their differentiation into tumor specific CD54+ neutrophils which suppressed refractory gastric cancer^58^. Conversely, deletion of *Yap/Taz* in neutrophils impaired their differentiation into CD54+ tumor specific neutrophils and reduced their antitumor activity, leading to accelerated gastric cancer progression^58^. Similarly, TAZ plays a role in the polarization of immune suppressive regulatory T-cells (Treg cells) in mice. Endogenous or experimental activation of TAZ in naïve CD4 + T-helper cells inhibited their differentiation into Treg cells but promoted the development of Th17 cells, a subtype of proinflammatory effector helper T-cells (Fig. 4A)^59^. Thus, this function of TAZ is tumor suppressive as it activates immune surveillance (Fig.4A). Mechanistically, TAZ activated the Th17-specifying transcription factor RORrγT and promoted the proteasomal degradation of the Treg master regulator FOXP3^59^. Thus, TAZ promotes the differentiation of Th17 cells at the expense of Treg cells. Notably, the Th17-inducing function of TAZ is independent of the canonical Hippo pathway transcription factors TEAD1-4: overexpression of TEAD1 acted antagonistically by sequestering TAZ and preventing the binding to RORγT and FOXP3, thereby enabling excessive Treg cell differentiation^59^. Because downregulation of TAZ activity is sufficient to increase the number of Treg cells in mice^59^, systemic inhibition of TAZ may promote tumor growth by suppressing antitumor immunity responses.

### Tumor suppressing functions of YAP/TAZ in peritumoral cells

Normal cells can activate an endogenous antitumorigenic defense mechanism, known as cell competition, which can restrain tumor development by direct competition between normal and malignant cells (Fig.4B)^60,61^. Cell competition refers to the phenomenon whereby “fitter” cells (also known as winner cells) cause the elimination of neighboring “weaker” or abnormal cells (also known as loser cells). In normal tissues this increases the overall health of the tissue by eliminating weak, damaged, or premalignant cells and replacing them with healthier normal cells. However, malignant cells can hijack this mechanism which then promotes tumor growth and survival at the expense of adjacent normal cells (Fig.4B)^60,61^. The relative level of YAP/TAZ between cells is a factor that determines whether a cell becomes a winner or loser: cells expressing higher levels of YAP/TAZ become winner cells while cells expressing relatively lower levels become loser cells^62^. Thus, activation of YAP/TAZ in cancer cells increases their relative fitness and drives tumor growth. Interestingly, normal hepatocytes surrounding liver tumors in mice activated YAP/TAZ, which elevated their competitiveness and restrained tumor growth^62^. This endogenous activation of YAP/TAZ in normal hepatocytes was not sufficient to halt the growth of liver tumors, but enough to restrain tumor growth because deletion of *Yap/Taz* in normal hepatocytes surrounding liver tumors exacerbated tumor growth^62^. Conversely, experimental hyperactivation of YAP/TAZ in peritumoral hepatocytes by knockout of *Lats1/2* or by conditional overexpression of *YAP*, triggered the elimination of early liver tumors and melanoma-derived metastases in the mouse liver^62^. Importantly, the survival of those tumor cells depended on the relative activity of YAP and TAZ in tumor cells versus surrounding parenchymal cells because deletion of *Yap/Taz* specifically in tumor cells suppressed tumor growth, but simultaneous deletion of *Yap/Taz* in tumor cells and parenchymal hepatocytes abolished cell competition and permitted tumor growth^62^. While it is not yet known whether similar tumor suppressor mechanisms take place in humans, human peritumoral hepatocytes also activate YAP in the presence of liver cancer but not in normal livers^62^. It is thus likely that cell competition is also relevant for the development of liver cancer in humans. Similar mechanisms may also operate in other organs such as the pancreas and brain^63,64^. In the pancreas, Ras^V12^ expressing cells can be apically extruded from the epithelium through cell competition with surrounding normal cells^63^, while in the brain differential expression of YAP in glioma cells leads to clonal dominance of tumor cells expressing higher levels of YAP and the induction of apoptosis in cells expressing lower levels of YAP^64^. These findings imply that, in some cases, systemic inhibition of YAP/TAZ-TEAD may dampen tumor suppressive cell competition thereby elevating tumor cell fitness and viability.

## Strategies for YAP/TAZ-TEAD inhibition in cancer therapy

**Fig. 5:**
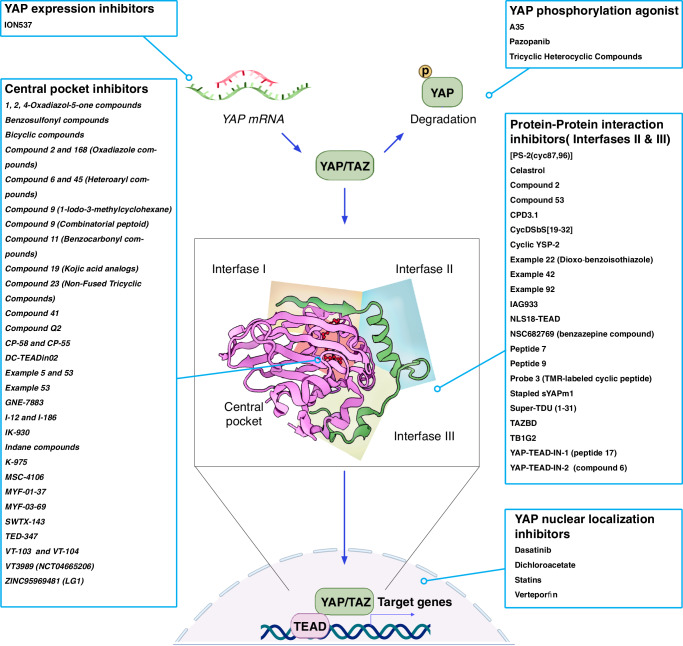
Small molecule YAP/TAZ-TEAD inhibitors. Several approaches and inhibitors have been developed to target YAP/TAZ, their interaction with TEAD transcription factors, and the transcriptional output of the Hippo pathway. This figure lists some of the inhibitors used to inhibit YAP/TAZ-TEAD transcriptional activity and their mode of action, which include promoting degradation of mRNA encoding for *YAP*, inducing YAP/TAZ protein degradation, interfering with the binding of YAP/TAZ to TEAD transcription factors and blocking YAP/TAZ nuclear localization.

Several strategies are being pursued to inhibit YAP/TAZ function, including the development of small molecule and RNAi-based inhibitors (Fig. 5)^6,8,65^. The first generation of YAP/TAZ inhibitors, such as Verteporfin, aimed at blocking the binding of YAP/TAZ to TEAD1-4, their canonical transcription factors^65^. However, Verteporfin and other inhibitors such as celastrol and narciclasine, are multi-target drugs that affect other non-YAP mediated processes such as autophagy and TNF signaling^66–68^. A new generation of TEAD inhibitors competitively bind to a conserved hydrophobic palmitate-binding pocket in TEAD proteins^6,8,65^. TEAD proteins auto-S-palmitoylate^69^, and blocking this activity causes TEAD protein instability and prevents their interaction with YAP/TAZ thereby reducing the transcriptional output of the Hippo pathway^8,65^. Another new class of small molecule YAP/TAZ inhibitors is based on a dihydrobenzofurane scaffold and directly inhibit the protein-protein interaction between YAP/TAZ and TEAD by binding to the Ω-loop pocket of TEADs^65^. Finally, Ionis Pharmaceuticals uses antisense oligonucleotide technology to deplete *YAP* mRNA (NCT04659096). Preclinical tests showed that some of these newer YAP/TAZ-TEAD inhibitors have good target specificity, cell penetration, and toxicity profiles, making them candidates for clinical testing. Which of these different approaches shows the best efficacy for cancer treatment is not yet known and may be different for different types of cancers or cancers with different mutations.

### Targeting Hippo pathway mutant cancers

One of the key hurdles in the use of inhibitors against YAP/TAZ-TEAD in cancer therapy lies in identifying the specific types of cancer that respond to YAP/TAZ-TEAD inhibition. Patients with tumors that are driven by YAP/TAZ activation due to mutations in Hippo pathway components may be the most suitable candidates for treatment with YAP/TAZ-TEAD inhibitors. A cancer with a high frequency of mutational YAP/TAZ-TEAD activation is malignant pleural mesothelioma, an asbestos-induced lung tumor where forty percent of patients have deletions or loss-of-function mutations in *LATS1*, *NF2, RASSF1, or SAV1*^70^. Importantly, mesothelioma cells are addicted to YAP/TAZ-TEAD activity for cell proliferation and survival and thus sensitive to YAP/TAZ-TEAD inhibitors^71^. Treating mouse models for mesothelioma based on human cell line xenografts or genetically induced orthotopic mesothelioma with YAP/TAZ-TEAD inhibitors caused tumor regression^2–7,72–76^. However, tumor elimination was generally not complete and tumor regrowth was observed in cases that reported longer term treatment^72,77^. The mechanism by which mesothelioma cells become resistant to the YAP/TAZ-TEAD inhibitors is not yet known. These data show efficacy against mesothelioma but indicate that a monotherapy may not be sufficient to cause significant and long-lasting clinical benefit.

Phase 1 clinical trials are currently assessing different YAP/TAZ-TEAD inhibitors in patients with mesothelioma and other cancers harboring *NF2/LATS1/LATS2*-mutations and tumors with functional *YAP/TAZ* fusions or otherwise elevated YAP/TAZ levels (Fig.5). Vivace therapeutics recently reported the first clinical data of using their YAP/TAZ-TEAD inhibitor VT-3989 (ClinicalTrials Identifier: NCT04665206) in patients with mesothelioma and other cancers with mutations in NF2^73^. They observed a partial response in 7 of 69 patients with refractory solid tumors (43 with mesothelioma, 9 with meningioma, and 17 with other solid tumors), while the rest showed stable disease. From those 7 responders, 6 had refractory mesothelioma (two *NF2* mutant, three *NF2* wild type and one unknown) and 1 had an *NF2* mutant sarcoma. These results are encouraging and demonstrate efficacy against a cancer that is otherwise difficult to treat. Another YAP/TAZ-TEAD inhibitor in clinical trial is IK-930 from Ikena (ClinicalTrials Identifier: NCT05228015). This trial is aimed at testing the safety, tolerability, pharmacokinetics, pharmacodynamics, and antitumor activity of IK-930 in patients with mesothelioma. An antisense oligonucleotide from Ionis Pharmaceuticals to target *YAP* mRNA is in Phase 1 trial in patients with neoplasms or advanced primary, recurrent or metastatic solid tumors (ClinicalTrials Identifier: NCT04659096). A Phase 2 study involves dose expansion in patients with molecularly selected advanced solid tumors. Ionis’ preclinical data demonstrated efficacy in inhibiting the proliferation of xenograft models of head and neck tumors, hepatocellular carcinoma, epidermoid carcinoma, and squamous cell carcinoma^78^. Thus, the preclinical data and the first data from these clinical trials show promising results with mesothelioma and other cancers.

### Non-Hippo pathway mutant cancers

A key question in developing YAP/TAZ inhibitors is to what extent YAP/TAZ-TEAD inhibition can show clinical benefit in patients with tumors that are not driven by mutations in Hippo pathway components and how to identify responding patients. YAP/TAZ are hyperactivated in various cancers, but it is largely unknown whether such cancer cells require YAP/TAZ for their survival or other cancer cell phenotypes. To address this, it will be important to test the efficacy of YAP/TAZ-TEAD inhibitors in various preclinical cancer models of YAP/TAZ-activated tumors that bear and do not bear Hippo pathway mutations. Extrapolating from the discussion in the previous section, however, simply measuring the effect of YAP/TAZ-TEAD inhibitors on cell proliferation in 2D or even 3D monocultures of cancer cells may not reliably predict efficacy in cancer patients. Rather, preclinical efficacy testing may require animal models that recapitulate the diversity of cancer associated cell types and cell-cell interactions like cell competition. Profiling of different cancers treated with YAP/TAZ-TEAD inhibitors may then uncover signaling pathways and cell states that correlate with YAP/TAZ activation and more importantly with their sensitivity to YAP/TAZ-TEAD inhibition. Such molecular characterization may identify potential biomarkers and genetic signatures that can predict the therapeutic response of YAP/TAZ-TEAD inhibitors and guide patient stratification.

### Combination therapy and drug resistance

Emerging evidence shows that YAP/TAZ activation contributes to therapy resistance in cancer cells through various mechanisms^79^. In human pancreatic ductal adenocarcinoma, YAP/TEAD2 complex with E2F in response to loss of oncogenic RAS signaling and induce cell proliferation and survival^80^. In melanoma mutant for BRAF and KRAS, YAP/TAZ activity promotes resistance to EGFR, KRAS^G12C^, and MEK inhibitors^79^. YAP can promote lung cancer cell survival and therapy resistance by activating the expression of MRAS, which can reactivate MAPK signaling in the absence of KRAS G12C signaling^81^. YAP/TAZ can also promote drug resistance by inducing the expression of drug efflux transporters, such as ABCG2 and MDR1, which allow lung cancer cells to pump harmful substances to the extracellular space, thus reducing the efficacy of the therapeutic drug^82^. In addition, YAP can also compensate for the absence of KRAS by converging with the transcription factor FOS to drive the KRAS-mediated transcriptional program in colon cancer^83^. Thus, combination therapies with YAP/TAZ-TEAD inhibitors may increase the efficacy of targeted and chemotherapies by reducing therapy resistance. YAP/TEAD signaling can also induce a senescence-like dormant state that allows cancer cells to resist the effect of different inhibitors, such as EGFR/MEK inhibitors^84^. These dormant cells are known as cancer persister cells, and are a subpopulation of resistant cancer cells capable of surviving initial targeted or chemotherapy treatment by entering into a dormant state and giving rise to recurrent disease^85^. YAP/TAZ can promote a persister cell state by activating a transcriptional program that reprograms cancer cells into a transient progenitor cell state characterized by p21 expression and reduced proliferation^86,87^. At least in some cases, pharmacological inhibition of YAP/TEAD can deplete dormant cells by inducing apoptosis^84^. However, the function of YAP/TAZ in persister cell biology is complex and might be context dependent: some studies indicate that YAP/TAZ activation maintains cells in a persister state^84,86,87^, while others show that YAP/TAZ activation is crucial for persister cell survival and proliferation upon resuming the cell cycle^88,89^. Nevertheless, even though the role of YAP/TAZ in persister and dormant cell fate is an emerging area of investigation, these findings present the intriguing possibility that inhibiting YAP/TAZ-TEAD activity may prevent cancer recurrence. Most importantly, these findings imply that combination therapies with YAP/TAZ-TEAD inhibitors may increase the efficacy of targeted and chemotherapies by reducing therapy resistance.

### YAP/TAZ-TEAD inhibitors and immunotherapy

YAP/TAZ-TEAD inhibition holds promise to enhance the efficacy of immune checkpoint inhibitors for cancer treatment. Inhibiting YAP/TAZ-TEAD can reduce the expression of immune checkpoint proteins, such as PD-L1, in cancer cells thereby diminishing immune evasion and enhancing T-cell anticancer function^90,91^. This may particularly benefit the treatment of patients with “hot” tumors, which already have immune cell infiltration, by making them even more responsive to immune checkpoint inhibition. Moreover, in “cold” tumors, which are characterized by poor immunogenicity, YAP/TAZ-TEAD inhibition may increase the expression of major histocompatibility complex (MHC) molecules, essential for presenting tumor antigens to adaptive immune cells and initiating an antigen-specific anti-tumor immune response^92^. Consequently, YAP/TAZ-TEAD inhibition may convert immune cold tumors into a more immune-responsive state, potentially boosting their sensitivity to immune checkpoint inhibitors and other immunotherapies. Moreover, the effects of YAP/TAZ-TEAD inhibition may cooperate in activating T-cell function because their inhibition blocks the tumor-protecting function of Treg cells^30,31^. However, Inhibiting YAP/TAZ-TEAD may also suppress neutrophil anti-cancer activities^59^. Further research is necessary to fully understand the benefits and limitations of combining immune therapy with YAP/TAZ-TEAD inhibition and to identify optimal treatment strategies for specific cancer types. Nonetheless, targeting YAP/TAZ activity represents a promising avenue for inducing T-cell activation and infiltration in poorly immunogenic tumors to enhance the effectiveness of immune therapy.

## Challenges for YAP/TAZ-TEAD inhibition in cancer therapy

### Tumor suppression versus tumor promotion

One of the major challenges in predicting the treatment effects of systemic YAP/TAZ-TEAD inhibition lies in our inability to predict the net effect on tumors of inhibiting their tumor-promoting and tumor-suppressing functions in different cellular compartments. In some cases, systemically inhibiting YAP/TAZ in cancer and associated cells may synergistically attack cancer cells for example by inhibiting a cell proliferation function of YAP/TAZ in cancer cells^6,65^ and by activating tumor suppressive cytotoxic T-cells^30,31,33,34^. However, in other cases, systemic YAP/TAZ-TEAD inhibition may trigger opposing effects in different cellular compartments, making outcome prediction challenging. For example, the deletion of *Yap/Taz* in cancer cells of mouse models for hepatocellular carcinoma caused complete tumor regression but the deletion of *Yap/Taz* in peritumoral hepatocytes enhanced tumor growth^62^. Surprisingly, tumors were still able to grow when *Yap/Taz* were simultaneously deleted in tumor cells and peritumoral hepatocytes. This example shows that it cannot naively be assumed that a cell-autonomous dependence of cancer cells on YAP/TAZ for their survival automatically translates into therapeutic efficacy when YAP/TAZ are systemically inhibited. Consequently, testing the effects of systemic YAP/TAZ inhibition and inhibition in different cellular compartments is crucial to determine safety, efficacy, and optimal strategies to employ YAP/TAZ-TEAD inhibitors for cancer treatment.

### Predicting efficacy

How can we better predict outcomes and identify patients who will benefit from YAP/TAZ-TEAD inhibition? Various approaches, such as genomic and transcriptional profiling, functional xenograft assays, and biomarker identification, can assist in stratifying potential patients. However, these methods have limitations when identifying suitable candidates. While genomic and transcriptional profiling can reveal YAP/TAZ activation in cancer cells, it may overlook activation in remote cells with anti-tumoral functions targeted by the inhibitors but not sampled by biopsies. Additionally, identifying effective biomarkers for patient stratification is challenging because YAP/TAZ activation in both tumor and non-tumor cells may drive similar gene signatures, regardless of their pro- or anti-tumor characteristics. Patient-derived xenograft assays (PDX) are considered the gold standard for preclinical studies, yet their prognostic ability for YAP/TAZ-TEAD inhibitors is limited due to the absence of immune cells and an artificial tumor microenvironment, which play significant roles in YAP/TAZ’s pro- and anti-tumoral functions. Hence, clinical trials should integrate data from these preclinical methods with comprehensive patient profiling to establish correlations between treatment response, patient characteristics, and potential predictive factors. Such integrated approaches can refine prediction and stratification strategies for the effective use of YAP/TAZ-TEAD inhibitors in cancer therapy.

### Adverse side-effects

Finally, while targeting the Hippo pathway for cancer therapy holds promise, the normal functions of YAP and TAZ in adult tissue homeostasis pose concerns for potential side effects of systemic YAP/TAZ-TEAD inhibition. One major problem may be the potential damage to the kidney. Loss-of-function experiments in mice showed that deletion of *Yap* in podocytes resulted in progressive renal failure due to increased apoptosis and gradual podocyte depletion^93^ Thus, YAP/TAZ-TEAD inhibition per se may pose risks but maybe more importantly, it may also compound the risk of renal failure for patients undergoing standard of care therapy. For example, radiation and chemotherapy can induce kidney damage and this damage may be exacerbated by YAP/TAZ-TEAD inhibition^93^. Also, patients with compromised immune systems might experience exacerbated immunosuppression due to YAP/TAZ’s immune regulatory roles. In addition, YAP/TAZ inhibition might disrupt the delicate balance between tissue turnover, repair and function, leading to impaired organ function or regenerative capacity^94^. For instance, radiation and chemotherapy can deplete intestinal stem cells and cause intestine injury and YAP/TAZ-TEAD inhibition might exacerbate intestinal damage due to the requirement of YAP for stem cell renewal and intestine regeneration^94^. Thus, these examples show that careful consideration is essential when using YAP/TAZ inhibitors, especially in patients with multifaceted medical conditions.

## Outlook, and future perspectives

While targeting the Hippo pathway as an anticancer therapeutic shows immense promise, it is not without challenges. An increasing body of experimental and clinical work suggest that patients suffering from several types of cancers may benefit from YAP/TAZ-TEAD inhibition. However, beyond tumors with mutations in Hippo pathway components, it is not clear which other human cancers may respond to YAP/TAZ-TEAD inhibitors. Fortunately, growing evidence support the idea that YAP activation promotes therapy resistance in various types of cancer. Thus, combination therapies using YAP/TAZ-TEAD inhibitors with other standard-of-care drugs may overcome therapy resistance. Therefore, in light of the potential benefits, we anticipate that systemic YAP/TAZ-TEAD inhibition will lead to the development of novel and increasingly effective pharmacological approaches.
